# Making supplemental information more accessible

**DOI:** 10.1186/s13059-018-1609-8

**Published:** 2018-12-21

**Authors:** Barbara Cheifet

**Affiliations:** New York, USA

## Abstract

Supplemental information is difficult to organize, review, and understand. *Genome Biology* has listed some new recommendations for the organization of supplemental data.

Scientists everywhere agree that supplementary material can be a problem [1, 2]. Reviewers often find it difficult to review all of the presented data, especially if information is tucked into poorly labeled tables or figures. Readers might struggle to find a specific figure or method that they wish to reproduce. Authors may not find it easy to know what information should be included. Very few standards exist to explain how supplemental material should be organized, and as a result, the formatting of supplements varies drastically from one published manuscript to the next. Feedback that we’ve received from the genomics community shows that reviewers and readers are strongly in favor of having supplemental data presented in a more formal and standardized format. Nevertheless, no author wants to have to reformat their (sometimes extensive) supplements upon acceptance or submission in order to fit with a particular journal’s guidelines, unless these guidelines were to be standardized across the majority of publishers.

Last year, *Genome Biology* published an Opinion article that put forward a structure for supplementary materials that supported reproducibility [3]. Since this article was published, our editorial team has been thinking about how we could implement some of the changes suggested by Greenbaum et al. Specifically, we wanted to establish some guidelines and recommendations for formatting supplemental data and materials that would make this content accessible to readers and reviewers, but without placing too much additional burden on our authors.

As datasets become larger and figures become more extensive, we feel that some form of structure should be suggested for supplements. Otherwise, potentially interesting findings may be lost, hidden, or not properly reviewed. Therefore, we are strongly in favor of authors’ formatting their supplemental files along the guidelines proposed by Greenbaum and colleagues.

Some of the suggestions in the Opinion article are potential ‘quick fixes’. For example, manuscripts that contain a single large ‘Additional file’ might benefit from the inclusion of a table of contents at the beginning of the file. This is a simple addition that would not require much effort from the authors, but which would make it much easier for reviewers and readers to find the particular supplemental figure or note that they are looking for.

Other suggestions from Greenbaum and colleagues would require the authors to expend more effort. The Opinion proposes a hierarchical structure for the supplement, in which supplement subheadings would be the same as the main subheadings of the manuscript. Any supplemental data relevant to a particular manuscript subheading would be found under the corresponding supplement subheading. This information could include, but would not be limited to, additional text or figures that aren’t part of the main story (such as negative results) or workflows relevant to specific figures or experiments. In theory, we think that this is a great idea as it clearly organizes the supplement in a way that is easy to follow. However, we recognize that this is not feasible for all types of submissions, and it does require some advanced planning and effort by the authors.

Many of our authors include ‘Supplemental methods’ as part of the supplement. In general, we prefer the majority of methods used in the paper to be included in the main ‘Methods’ section of the manuscript. *Genome Biology* also requires all software code or scripts to be deposited in a publicly available repository, with appropriate license information and citations included. We realize, however, that there may be additional methods, or details about the code or methods, that could be usefully included in additional files. For example, authors may wish to include tables of reagents used. Where information relating to methods is not included in the main ‘Methods’ section of the manuscript, it is even more crucial that these details can be found easily by the reader or user who wishes to replicate an analysis described in the paper.

What we have come up with, and what has now been posted in the ‘Submission guidelines’ section of our website, is a list of recommendations for the structuring of supplements. We are not requiring that our authors follow these guidelines, but we want to highlight them because we do feel that manuscripts will be more accessible and reproducible if authors think about the organization of the supplement while in the early stages of writing their paper. In the age of ‘big data’, we also think that it is part of the journal’s responsibility to step forward and encourage change so that data are not in danger of being lost.
